# Amurensin H, a Derivative From Resveratrol, Ameliorates Lipopolysaccharide/Cigarette Smoke–Induced Airway Inflammation by Blocking the Syk/NF-κB Pathway

**DOI:** 10.3389/fphar.2019.01157

**Published:** 2019-10-04

**Authors:** Yannan Fan, Ziqian Zhang, Chunsuo Yao, Jinye Bai, Hui Yang, Pei Ma, Yiyao Fan, Shuyi Li, Jiqiao Yuan, Mingbao Lin, Qi Hou

**Affiliations:** State Key Laboratory of Bioactive Substance and Function of Natural Medicines, Institute of Materia Medica, Chinese Academy of Medical Sciences and Peking Union Medical College, Beijing, China

**Keywords:** amurensin H, chronic obstructive pulmonary disease, airway inflammation, spleen tyrosine kinase, nuclear factor κB

## Abstract

Amurensin H, a resveratrol dimer derived from Vitis amurensis Rupr, has several biological effects, including anti-inflammatory and antioxidant activities. Studies have found that amurensin H attenuated asthma-like allergic airway inflammation. However, its protective activity on chronic obstructive pulmonary disease (COPD) airway inflammation is not fully explored. The present study used a lipopolysaccharide (LPS)/cigarette smoke–induced mice model and an LPS-stimulated THP-1–derived macrophages model to measure the lung tissue’s morphology changes. The results showed that amurensin H ameliorated the histological inflammatory alterations in the lung tissues, leading to a decrease in the expression of interleukin 6 (IL-6), IL-17A, tumor necrosis factor α (TNF-α), and interferon γ in bronchoalveolar lavage fluid. Amurensin H also significantly inhibited the release of IL-1β, IL-6, IL-8, and TNF-α in LPS-stimulated THP-1–derived macrophages. Furthermore, amurensin H markedly inhibited the expressions of p-Syk, nuclear factor κB (NF-κB), and p-NF-κB both *in vivo* and *in vitro*. Results from cotreatment with Syk inhibitor BAY61-3606 and NF-κB inhibitor BAY11-7082 *in vitro* revealed that amurensin H’s protective effect against airway inflammation could be due partly to the inhibition of the Syk/NF-κB pathway. These findings suggest that amurensin H shows therapeutic effects on COPD airway inflammation, and inhibiting the Syk/NF-κB pathway might be part of its underlying mechanisms.

## Introduction

Chronic obstructive pulmonary disease (COPD), a disease characterized by chronic airway inflammation and persistent airflow limitation, is the third most common noncommunicable cause of death worldwide (Collaborators of G.C.O.D, 2017). As a heterogeneous disease, its pathological processes are featured by chronic bronchitis and emphysema, both of which lead to impaired lung function. The association of chronic airway inflammation with COPD always initiates or worsens systemic comorbidity, resulting in increased morbidities and mortality and rising economic and social burdens (Cavaillès et al., 2013). Thus, anti–airway inflammation could be regarded as an important choice for COPD treatment.

**Figure 1:**
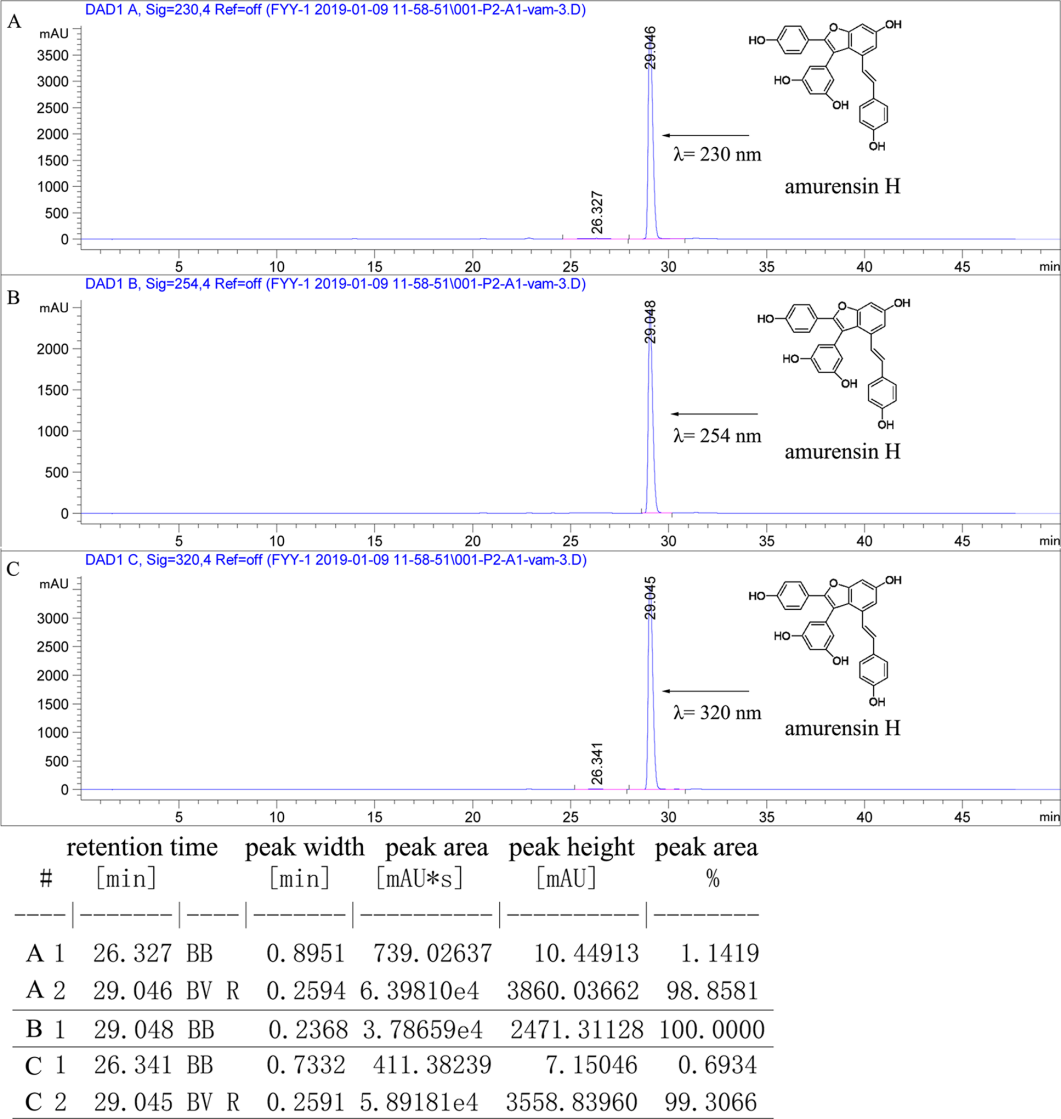
The purity of amurensin H was analyzed by HPLC chromatogram at 230 nm **(A)**, 250 nm **(B)**, and 320 nm **(C)**. The flow rate was 1.0 ml/min, and the injection volume was 10 µl.

Bronchodilators and steroids are current medications for COPD, while COPD subjects often develop resistance to corticosteroid anti-inflammatory treatments (Barnes, 2013). However, there is no safe and effective therapy to modulate airway inflammation in COPD progression; the search for new types of anti-inflammatory medicines remains a priority. In recent years, herbal medicine has been used as an adjunct therapy for COPD treatment, potentially attenuating airway inflammation and improving the quality of life for COPD patients. But there is lack of randomized controlled clinical trial data for treatment of COPD with herbal medicine that demonstrates effectiveness (Chen et al., 2014). *Vitis amurensis* Aupr., a species of wild-growing grape originating from east Asian, has been used as a traditional Chinese herb for hundreds of years to treat pain and cancer. Recent studies revealed that roots and stems of *V. amurensis* possessed antioxidant, anti-inflammatory, antibacterial, and cardioprotective activities and contained various oligomers of resveratrol (Huang et al., 2001; Chen et al., 2018). Resveratrol (3,5,4′-trihydroxy-*trans*-stilbene) has demonstrated its ability to alleviate inflammation in airway diseases and suppress cigarette smoke (CS)–induced oxidative lung inflammatory injury (Wood et al., 2010; Knobloch et al., 2014). Amurensins, derivatives of resveratrol isolated from the roots of *V. amurensis*, also showed anti-inflammatory activities of amurensin *in vitro* (Huang et al., 2000). Amurensin H is a new resveratrol dimer, which was first isolated from *V. amurensis* Rupr. and was synthesized from resveratrol with an oxidative coupling reaction as a key step or directly synthesized from substituted stilbene (**Figure 1**) (Huang et al., 1999; Kraus and Gupta, 2009). Our previous studies have highlighted the capability of amurensin H to inhibit inflammatory responses and suppress the production of ROS induced by lipopolysaccharide (LPS)/ATP via caspase-1/interleukin 1β (IL-1β) pathway in mouse peritoneal macrophages; activities of MMP-9 in THP-1-derived macrophages and expression of ICAM-1 in ovalbumin-induced asthmatic mice were also downregulated, indicating amurensin H’s antioxidant and anti-inflammatory potential (Yang et al., 2010; Shi et al., 2012; Cao et al., 2014). Previous research also showed that oral administration of amurensin H had a potential antiallergic inflammation effect in OVA-induced mice and may work in the treatment of allergic airway inflammation (Li et al., 2006). Asthma and COPD both are airway diseases characterized by chronic airway inflammation. Moreover, amurensin H reportedly acts as an antiautophagy agent *via* regulating levels of Sirt1 and FoxO3 and suppressing oxidative stress in CS-induced autophagy model and shows a potential preventive effect in the progression of COPD (Shi et al., 2012). The purpose of this study herein is to investigate activities of amurensin H more extensively and deeply in COPD inflammation and examine the possible mechanisms.

COPD is a disorder characterized by an abnormal inflammatory immune response. Among several etiological factors of COPD, CS is considered as the leading risk factor, although a substantial portion of COPD sufferers is made up of nonsmokers (Eisner et al., 2010). Inhaled tobacco smoke brings about damage to the epithelial barrier, drives inflammatory cascades, generates tissue remodeling, and initiates the inflammatory progress of COPD (Shaykhiev and Crystal, 2014). Spleen tyrosine kinase, also known as Syk, plays crucial roles in both adaptive immune responses and innate immune responses (Yi et al., 2014). Amurensin H has been shown to be an ATP-competitive inhibitor of Syk *in vitro* (Jiang et al., 2014). Binding of ligand and cell membrane receptors that associate with transmembrane proteins with cytoplasmic domains containing immunoreceptor tyrosine-based activation motifs (ITAMs) leads to the recruitment of Syk. Syk is activated fully by phosphorylation or binding to ITAM *via* its tandem Src homology 2 (SH2) domains. Dual-phosphorylated ITAMs recruit Syk, engage Syk docks to ITAM, and undergo phosphorylation at different tyrosine residues, thus triggering kinase activation and downstream signaling (Mócsai et al., 2010; Buitrago et al., 2013; Mansueto et al., 2019). Besides, Syk also mediates signaling by novel classes of receptors that do not contain ITAM sequences. In its downstream signaling, Syk can activate the nuclear factor κB (NF-κB) pathway, which subsequently triggers the excessive production of chemokines and cytokines, leading to inflammatory responses (Lee et al., 2009). In light of the fundamental roles of Syk and NF-κB in airway inflammation, it is likely that the Syk/NF-κB pathway contributes to the progression of COPD.

Therefore, in this study, we hereby assessed the effect of amurensin H on COPD airway inflammation in an LPS/CS–induced murine model and an LPS-stimulated THP-1–derived macrophage model and then investigated its underlying mechanisms in Syk/NF-κB pathway.

## Materials and Methods

### Chemicals and Reagents

LPS (*Escherichia coli* 0111: B4), phorbol 12-myristate 13-acetate (PMA), BAY61-3606, and BAY11-7082 were purchased from Sigma-Aldrich (St. Louis, MO, USA). Dexamethasone sodium phosphate injection was purchased from Sinopharm Group Rongsheng Pharmaceutical Co., Ltd. (Jiaozuo, China). Amurensin H and roflumilast were prepared as before (Yang et al., 2010). The purity of amurensin H was greater than 98%, analyzed by analytical high-performance liquid chromatography (HPLC) on an Agilent 1260 HPLC/DAD-UV system equipped with a Capcell Pak C18 column (4.6 i.d. ×250 mm, S-5 µm; Osaka Soda Co., Ltd., Osaka, Japan), eluted with 80% methanol/water (ν = 1 ml/min, λ = 230, 254, 320 nm) at room temperature (**Figure 1**). Sodium chloride injection was purchased from Shandong Qidu Pharmaceutical Co., Ltd. (Zibo, China). Cigarette used in this experiment was Daqianmen (11 mg tar, 0.8 mg nicotine; Shanghai Tobacco Group Co., Ltd., Shanghai, China). RPMI-1640 and fetal bovine serum (FBS) were obtained from Gibco (NY, USA). All enzyme-linked immunosorbent assay (ELISA) kits were obtained from Biolegend (San Diego, CA, USA). Anti–phospho-Syk (Y323) (ab63515) was obtained from Abcam (Cambridge, UK). All other antibodies were obtained from Cell Signaling Technology (Boston, MA, USA): anti–β-actin (4970), anti-Syk (2712), anti-p65 (8242), anti–phospho-NF-κB p65 (Ser536) (3033S), anti-rabbit horseradish peroxidase (HRP)–conjugated secondary antibody (7074).

### Animals

Male Balb/c wild-type mice (6–8 weeks old, 20–22 g; Experimental Animal Center, Academy of Military Medical Sciences, China) were maintained under controlled conditions with a 12-h light/dark cycle, at a temperature of 21°C ± 2°C, and freely drinking and eating. All animal experiments were performed in accordance with the institutional guidelines at the Experimental Animal Center of the Institute of Materia Medica, Chinese Academy of Medical Sciences & Peking Union Medical College (Beijing, China), and conformed to internationally accepted ethical standards.

### LPS/CS-Induced Airway Inflammation and Treatment

**Figure 2:**
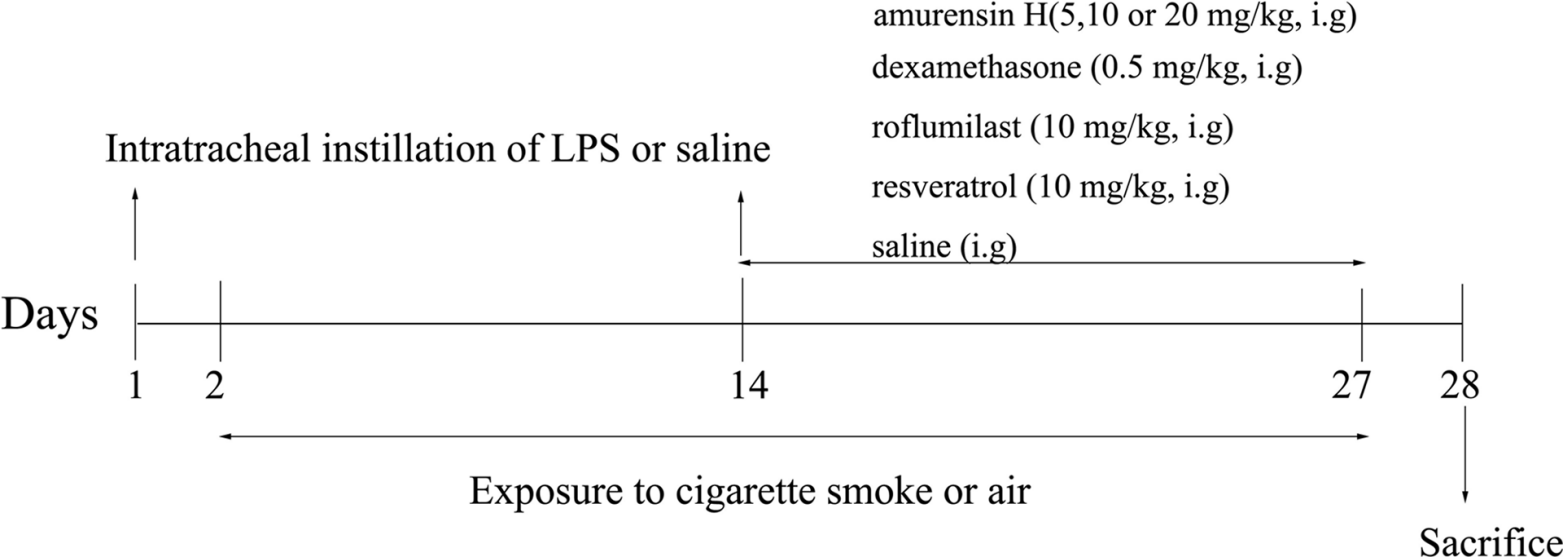
Timeline of the experimental protocol.

Mice were randomly divided into 8 groups: control group (n = 12), LPS/CS-induced mice model group (n = 14); amurensin H 5, 10, and 20 mg/kg group (n = 14 in each group); dexamethasone sodium phosphate injection group (DEX, n = 14); resveratrol group (RES, n = 14); and roflumilast group (RFST, n = 14). This experiment lasted for 28 days (**Figure 2**). Mice in the experimental groups were anesthetized and received intratracheal instillation of LPS (40 µg in 50 µl physiological sodium chloride solution) on days 1 and 14; animals in the control group were given saline solution in the same volume. From days 2 to 27 (except on day 14), mice were exposed to CS daily for 1 h in a microenvironment system specially designed for smoke inhalation by small animals (MBL-1; Institute of Materia Medica, Chinese Academy of Medical Sciences, Beijing, China); the conditions were controlled, with 250 to 300 ppm density of smoke particles let into the environment after lighting four cigarettes, the temperature of the environment was maintained at 25°C ± 2°C, and oxygen concentration exceeded 18%. All animals received an intragastric administration of vehicle or amurensin H (5, 10, and 20 mg/kg), dexamethasone sodium phosphate injection (0.5 mg/kg), phosphodiesterase 4 inhibitor roflumilast (10 mg/kg), and resveratrol (10 mg/kg) daily from days 14 to 27, 1 h before air or CS exposure. We calculated the final drug concentration administered of amurensin H (5, 10, and 20 mg/kg) and resveratrol (10 mg/kg) based on a dose we previously used (Xuan et al., 2014). The dose of roflumilast (10 mg/kg) was set at the same dose of medium dosage of amurensin H. The dose of dexamethasone (0.5 mg/kg) was calculated using the body surface area.

### Measurement of Cell Counts in Bronchoalveolar Lavage Fluid

Twenty-four hours after the last administration, mice were sacrificed. Bronchoalveolar lavage fluid (BALF) was collected by intratracheal instillation with a volume of 0.7 ml saline triply and then was centrifuged at 1500 rpm for 10 min at 4°C to separate cells from the supernatant. The supernatant was removed and stored at −80°C for enzyme-linked immunosorbent assays. The resulting cell pellets were resuspended with 0.5 ml 0.9% saline, and the total cell numbers, neutrophils, lymphocytes, monocytes, and eosinophils were counted using a hemocytometer (Mindray BC-5000 vet, Shenzhen, China). Typically, the recovery of BALF exceeded 85%, and the percentages of recovered fluid did not differ significantly among experimental groups.

### Histological Assessment of Lung Tissue

Lung tissues of mice were excised, fixed in 10% formalin solution, embedded in paraffin, and cut into 4-µm slides and then stained with hematoxylin-eosin (H&E) according to standard protocols. The grade of inflammatory reaction in the lungs was scored *via* analysis of inflammatory cell infiltration around the bronchus and vessels. The results were expressed as average on a four-point scale: grade 0, no observable inflammatory cell infiltration; grade 1, occasional cuff-like inflammatory cell infiltration; grade 2, infiltration of inflammatory cells in most of the veins/bronchus and 1 to 5 layer(s) of inflammatory cells; grade 3, obvious infiltration of inflammatory cells in most of the veins/bronchus and inflammatory cells greater than 5 layers. Histological analysis was performed in a single-blind fashion.

### Cell Culture, Stimulation, and Treatment

THP-1 cells were purchased from the national infrastructure of cell line resource (Beijing, China) and cultured in RPMI-1640 medium supplemented with 10% FBS, 2 mM l-glutamine, 0.05 mM 2-mercaptoethanol, and 100 U/ml penicillin and 100 µg/ml streptomycin. To differentiate THP-1 cells into macrophages, the cells were seeded into 96-well plates or six-well plates (1 × 10^6^ cells/ml), incubated for 48 h with phorbol 12-myristate 13-acetate (20 nM), then washed three times with phosphate-buffered saline, and incubated with RPMI-1640 for 4 days (Xu et al., 2011). THP-1–derived macrophages were pretreated with amurensin H (2.5, 5, and 10 µM), BAY61-3606 (an inhibitor of Syk, 0.5 µM), and BAY11-7082 (an inhibitor of NF-κB, 5 µM) for 1 h and then stimulated with LPS (1 µg/ml) for 24 h; macrophages in the control group were treated with the same dose of dimethyl sulfoxide. Cell supernatant in 96-well plates was used to detect the production of cytokines, and cells in six-well plates were used to undergo following experiments.

### Quantitative Reverse Transcription–Polymerase Chain Reaction Detection

Cultured cells were collected, and RNA was prepared using an RNA kit according to the manufacturer’s instructions (Transgen, Beijing, China). cDNA was synthesized using 6 µl of total RNA (300 ng) according to the Omniscript RT kit protocol (Qiagen, Germany) as described by the manufacturer. Commercial primers for Syk, p65, and β-actin (DHS57991, DHS445896, DHS938729) were purchased from XY biotech (Shanghai, China). Real-time polymerase chain reaction (PCR) was performed following the instructions of the top green quantitative PCR SuperMix (Transgen) in a real-time PCR machine (MYGO PRO, IT-IS, Ireland). Expression of target genes was corrected by the expression of β-actin housekeeping gene and calculated using the 2^−ΔΔCt^ method.

### Enzyme-Linked Immunosorbent Assay

Inflammatory cytokines in the BALF and cell culture supernatant were examined by mouse IL-6, IL-17A, interferon γ (IFN-γ) and tumor necrosis factor α (TNF-α) ELISA kits, human IL-1β, IL-6, TNF-α, and IL-8 ELISA kits according to the manufacturer’s instructions, respectively.

### Western Blotting Analysis

Thawed lung tissues were lysed in lysis buffer. In order to accelerate lysis, we crushed lung tissues in ice bath with a tissue homogenizer and vortexed the homogenates every 10 min (30 s each time, four times). The supernatants were collected by centrifugation at 12,000*g* for 10 min and assayed with BCA protein assay kit (Solarbio, Beijing, China). Cell lysates were prepared as described previously. Equal amounts of protein were separated by 10% sodium dodecyl sulfate–polyacrylamide gel electrophoresis and transferred onto polyvinylidene difluoride membranes (Millipore, USA), which were subsequently blocked with 5% skim milk in TBS-T (TBS+0.05% Tween-20) at room temperature for 1 h and then washed with TBST. The membranes were first probed with primary antibodies including anti–β-actin, anti-Syk, anti–phospho-Syk (Y323), anti-p65, and anti–phospho-NF-κB p65 (Ser536) separately overnight at 4°C. After washing, the membranes were incubated with anti-rabbit HRP-conjugated secondary antibody at room temperature for 2 h. Digital images were subsequently captured with a Chemical Imaging System, and ImageJ software was used for densitometric analysis.

## Statistical Analysis

Data are presented as mean ± SD. All statistical analysis was performed using GraphPad PRISM 7.00 (GraphPad, La Jolla, CA). The results were analyzed using one-way analysis of variance or Mann–Whitney *U* test, and *P* < 0.05 was considered statistically significant.

## Results

### Amurensin H Suppressed the Infiltration of Inflammatory Cells and Leukocytes Recruitment in the Lungs of LPS/CS-Induced Airway Inflammation in Mice

**Figure 3:**
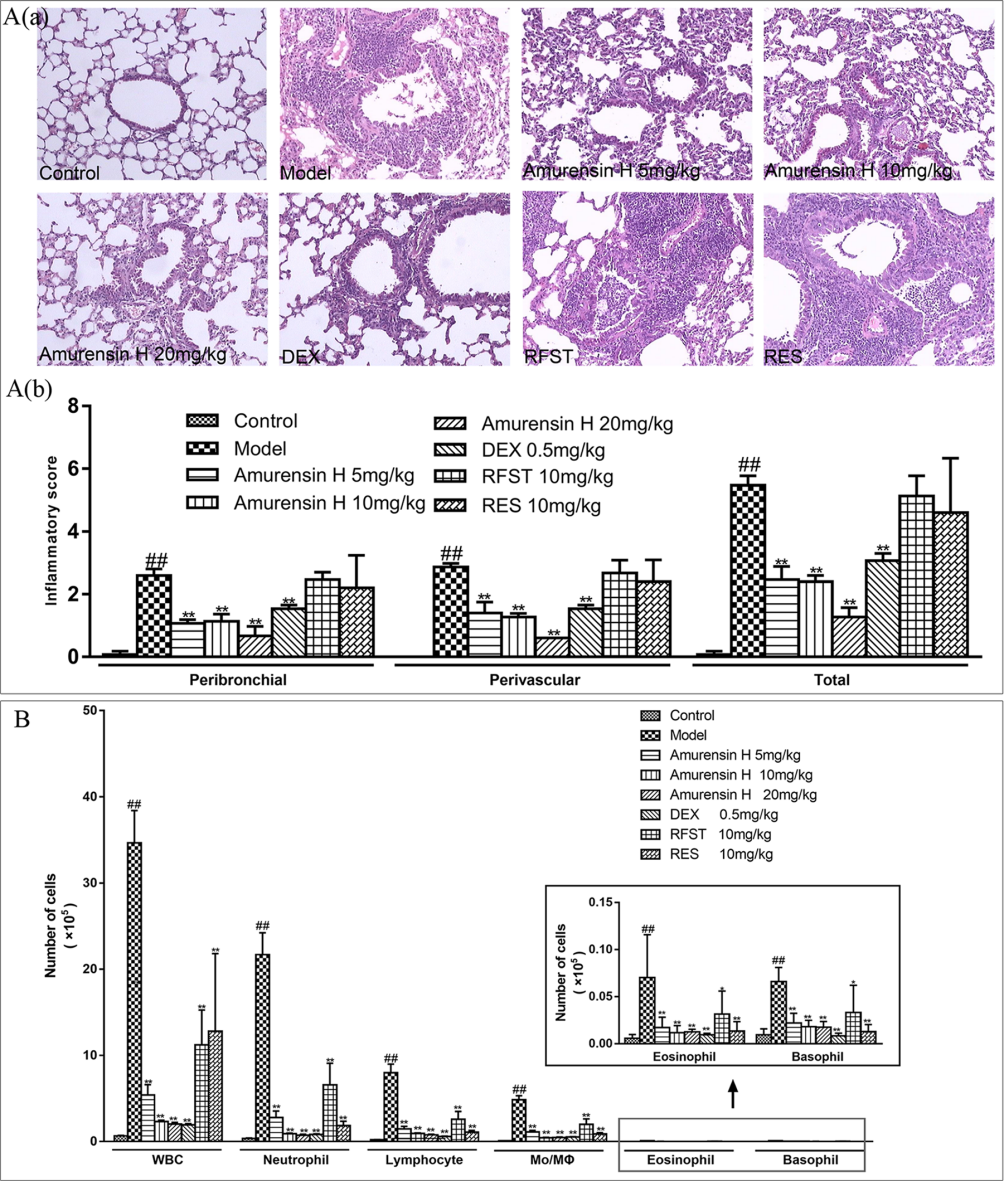
Effects of amurensin H on the inflammatory cell infiltration and leukocytes recruitment in LPS/CS-induced airway inflammation in mice. Mice were orally treated with vehicle, amurensin H, dexamethasone sodium phosphate injection, roflumilast, and resveratrol. **(Aa)** Representative images of H&E-stained lung sections from LPS/CS-induced mice and the various treatment strategies investigated (×100), demonstrating the extent of inflammatory cell infiltration within the bronchial and vascular wall. **(Ab)** Peribronchial and perivascular inflammation score from five fields/mouse section, where sections were scored based on cell infiltration around the bronchus and vessels on a scale of 0 (no observable inflammatory cell infiltration) to 3 (obvious infiltration of inflammatory cells in most of the veins/bronchus and inflammatory cells of more than five layers). n = 3 mice/group. **(B)** Total and differential leukocyte counts in BALF recovered in the airways of mice exposed to CS, n = 8 mice/group (except n = 5 in Control). Control, normal control mice; Model, LPS/CS-induced mice; DEX, mice treated with dexamethasone sodium phosphate injection (0.5 mg/kg); RFST, mice treated with roflumilast (10 mg/kg); RES, mice treated with resveratrol (10 mg/kg). Data are shown as mean ± SD; ^##^ *P* < 0.01 compared with the Control group; *P < 0.05 and **P < 0.01 compared with the Model group.

To evaluate the effect of amurensin H on airway inflammation in mice, H&E staining was used to assess the lung inflammatory changes, and an inflammatory score was used to quantify the inflammatory cell accumulations in peribronchial and perivascular sites. As shown in **Figure 3A**, LPS/CS-induced mice (model group) exhibited extensive infiltration of inflammatory cells into peribronchial and perivascular regions, which were significantly attenuated in amurensin H group (*P* < 0.01), as well as in the DEX group (*P* < 0.01). However, sections of mice treated with roflumilast and resveratrol also undergo heavy infiltration of inflammatory cells.

The levels of leukocytes in BALF were also measured (**Figure 3B**). In agreement with the histological appearance, LPS/CS-induced mice showed a significantly higher number of total leukocytes, neutrophils, lymphocytes, monocytes, macrophages, eosinophils, and basophils in BALF. When treated with amurensin H (5, 10, and 20 mg/kg) and DEX (0.5 mg/kg), counts of these leukocytes decreased. Comparably, the positive control roflumilast (a selective and long-acting inhibitor of the enzyme phosphodiesterase-4) and resveratrol (a derivative of stilbene) treatment also notably lessened the levels of total and differential leukocytes in BALF. Infiltration of inflammatory cells and the levels of leukocytes in BALF treated with amurensin H showed better outcomes than roflumilast and resveratrol treatment at the same dosage. However, with roflumilast and resveratrol treatment, there is only a significant decrease of leukocytes in BALF without tissue inflammatory cell infiltrations in the histological examination, this might because leukocytes infiltrated to alveolar spaces are susceptible to roflumilast and resveratrol treatments, histological changes might take more time, but the exact reason still needs to be explored. Positive control DEX, roflumilast, and resveratrol treatment also notably lessened the levels of total and differential leukocytes and cytokines in BALF.

### Amurensin H Inhibited the Production of IL-6, IL-17A, TNF-**α**, and IFN-**γ** in the BALF of LPS/CS-Induced Airway Inflammation in Mice

**Figure 4:**
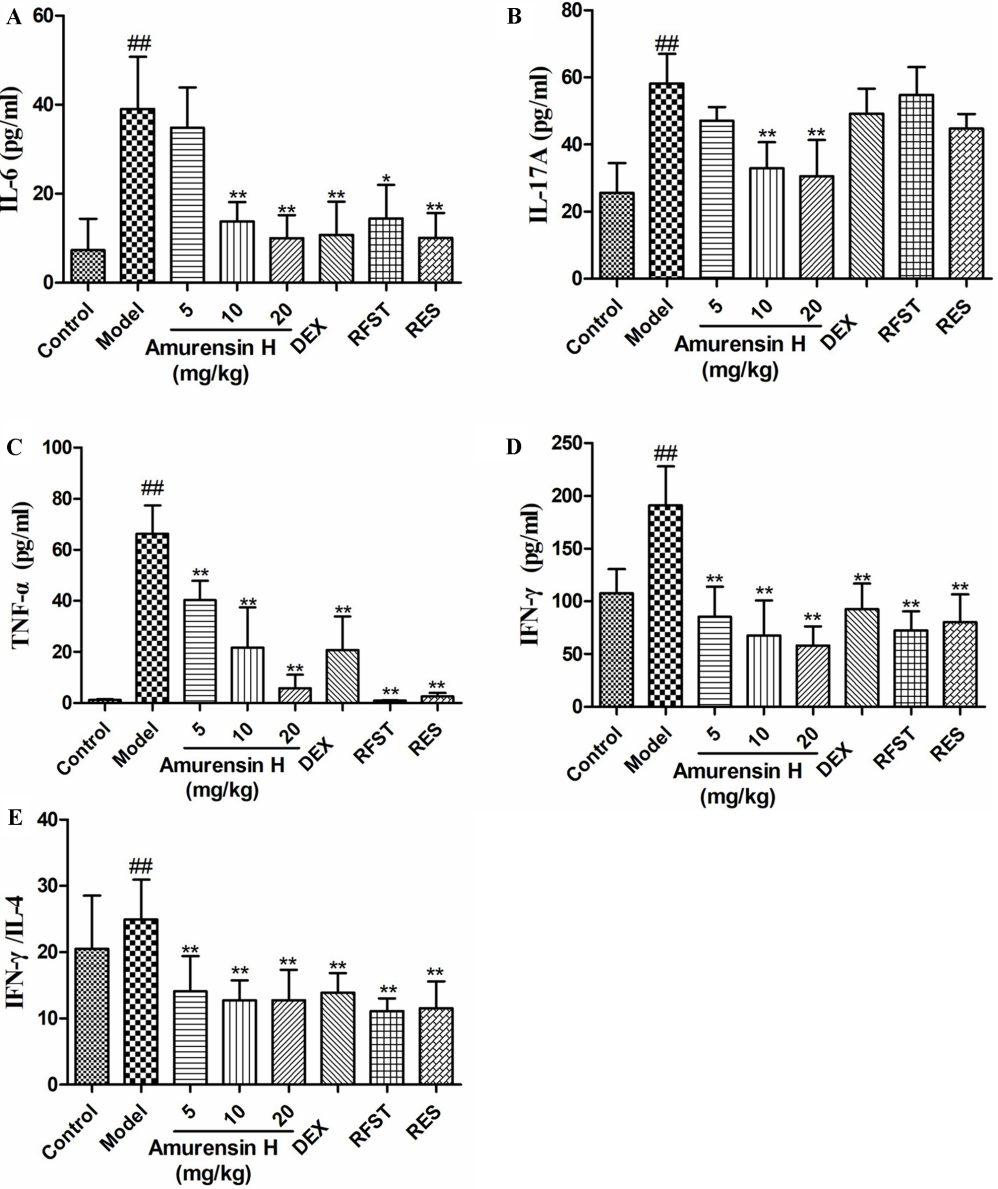
Effects of amurensin H on cytokines in the BALF of LPS/CS-induced mice. Mice were orally treated with vehicle, amurensin H, dexamethasone sodium phosphate injection, roflumilast, and resveratrol. Levels of IL-6 **(A)**, IL-17A **(B)**, TNF-α **(C)**, IFN-γ **(D)**, and ratio of IFN-γ/IL-4 **(E)** in the BALF from mice were detected by ELISA. Abbreviations: Control, normal control mice; Model, LPS/CS-induced mice; DEX, mice treated with dexamethasone sodium phosphate injection (0.5 mg/kg); RFST, mice treated with roflumilast (10 mg/kg); RES, mice treated with resveratrol (10 mg/kg). Data are shown as mean ± SD, n = 8 mice/group (except n = 5 in Control); ^##^ *P* < 0.01 compared with the Control group; *P < 0.05 and **P < 0.01 compared with the Model group.

Enzyme-linked immunosorbent assay was employed to measure the levels of inflammatory cytokines in BALF. Compared with the model group, amurensin H treatment significantly inhibited the production of IL-6 (**Figure 4A**), IL-17A (**Figure 4B**), TNF-α (**Figure 4C**), and IFN-γ (**Figure 4D**) in BALF and significantly decreased the ratio of IFN-γ/IL-4 (**Figure 4E**), showing a restoration of Th1 bias. Amurensin H’s inhibitory power of these inflammatory cytokines was dose dependent.

### Amurensin H Inhibited the Expression and Activation of Syk and NF-**κ**B p65 in the Lung Tissues of Mice Exposed to CS and LPS

**Figure 5:**
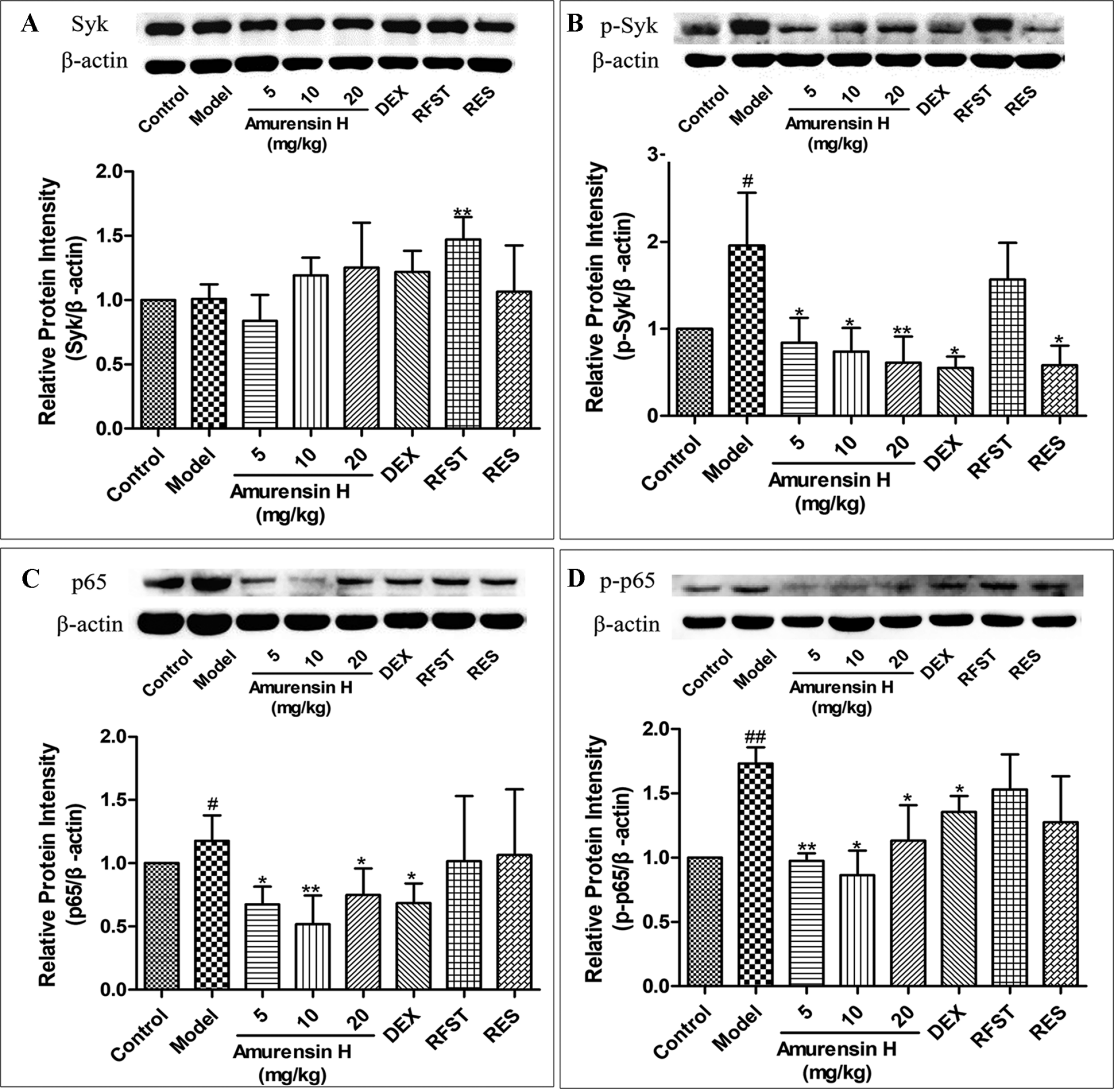
Expression and activation of Syk and NF-κB p65 in the lungs of LPS/CS-induced mice. Mice were orally treated with vehicle, amurensin H, dexamethasone sodium phosphate injection, roflumilast, and resveratrol. Effects on expression of Syk **(A)**, phosphorylated Syk(Y323) **(B)**, NF-κB p65 **(C)**, and phosphorylated NF-κB p65 **(D)** were detected by Western blotting. Representative Western blot showed amurensin H reduced phosphorylated Syk(Y323), p65, and p-p65 expression. Abbreviations: Control, normal control mice; Model, LPS/CS-induced mice; DEX, mice treated with dexamethasone sodium phosphate injection (0.5 mg/kg); RFST, mice treated with roflumilast (10 mg/kg); RES, mice treated with resveratrol (10 mg/kg). Data are shown as mean ± SD (n = 3). ^#^ *P* < 0.05 and ^##^ *P* < 0.01 compared with the Control group; *P < 0.05 and **P < 0.01 compared with the Model group.

Western blotting was used to investigate the effect of amurensin H on the expression of Syk (**Figure 5A**), p-Syk (**Figure 5B**), NF-κB (**Figure 5C**), and p-NF-κB (**Figure 5D**) in the lung tissues of LPS/CS-induced mice (**Supplementary Figures S1–S4**). As shown in **Figure 5**, compared with the control group, LPS/CS-induced mice (model group) showed a notable increase in p-Syk, NF-κB, and p-NF-κB expression (*P* < 0.05), but not in Syk. Amurensin H treatment significantly inhibited the p-Syk, NF-κB, and p-NF-κB alterations (*P* < 0.05 or *P* < 0.01), acting dose-dependently on p-Syk expression. Nevertheless, compared with the model group, there were no significant changes in Syk expression after amurensin H treatment. Collectively, the results suggest that amurensin H ameliorated airway inflammation by inhibiting the phosphorylation of Syk and then suppressing the expression and activation of its downstream inflammatory factor NF-κB p65. The positive control DEX only downregulated the expression of p-Syk and p65, resveratrol reduced the expression of p-Syk, whereas roflumilast had few effects on activation of Syk and NF-κB p65.

### Amurensin H Inhibited the Production of Inflammatory Cytokines in LPS-Stimulated THP-1–Derived Macrophages *in Vitro*

**Figure 6:**
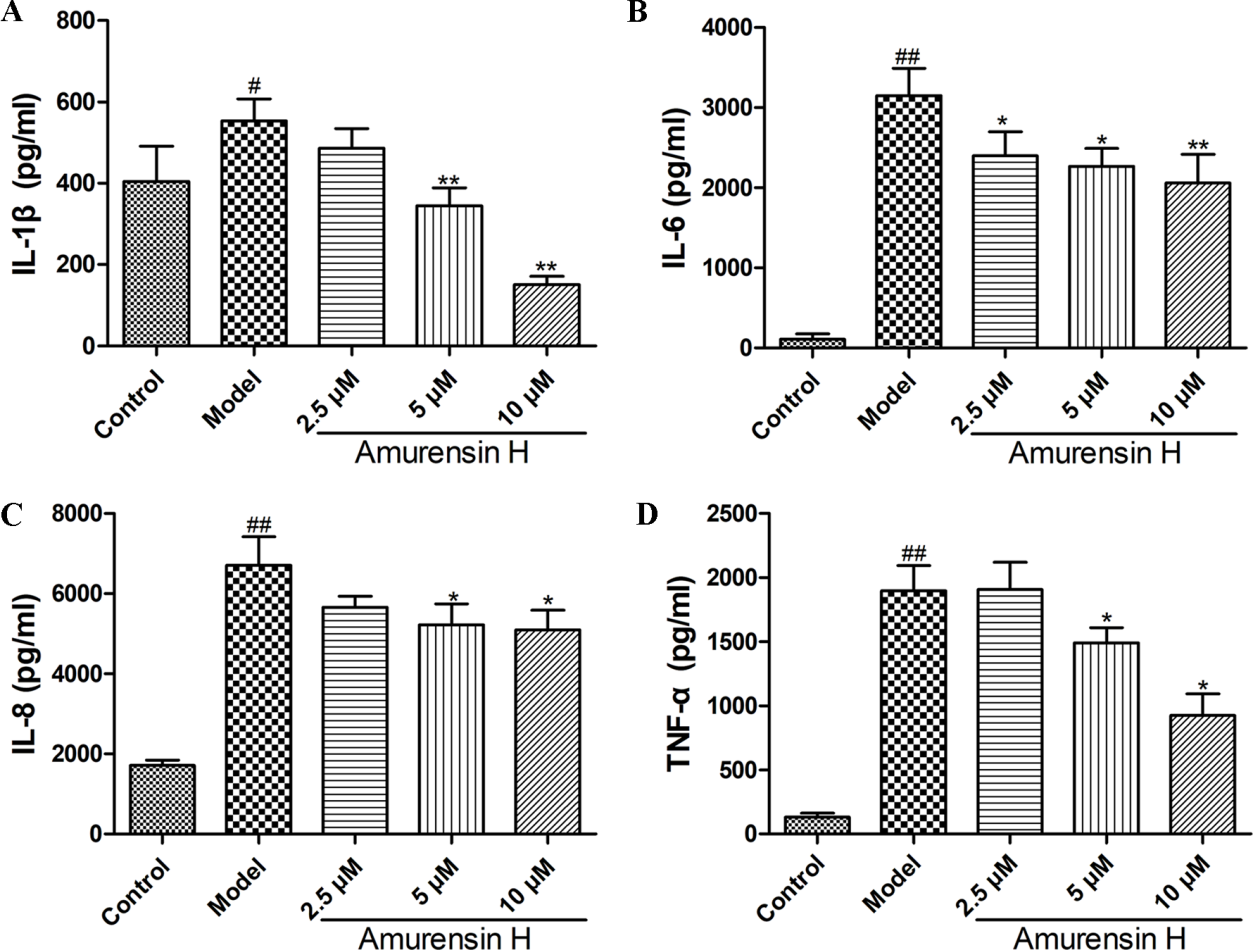
Levels of IL-1β **(A)**, IL-6 **(B)**, IL-8 **(C)** and TNF-α **(D)** in LPS-stimulated THP-1–derived macrophages treated with vehicle and amurensin H (2.5, 5, 10 µM) were detected by ELISA. Data are shown as mean ± SD of three to four independent experiments. ^#^ *P* < 0.05 and ^##^ *P* < 0.01 compared with the Control group; *P < 0.05 and **P < 0.01 compared with the Model group.

Lipopolysaccharide-stimulated THP-1–derived macrophages were used to analyze the anti-inflammatory effect of amurensin H *in vitro*. Compared to LPS-stimulated group, the production of IL-1β (**Figure 6A**), IL-6 (**Figure 6B**), IL-8 (**Figure 6C**), and TNF-α (**Figure 6D**) was significantly suppressed by amurensin H in a dose-dependent manner.

### Amurensin H Decreased the Expression of Syk and p65 in LPS-Stimulated THP-1–Derived Macrophages *in Vitro*

**Figure 7:**
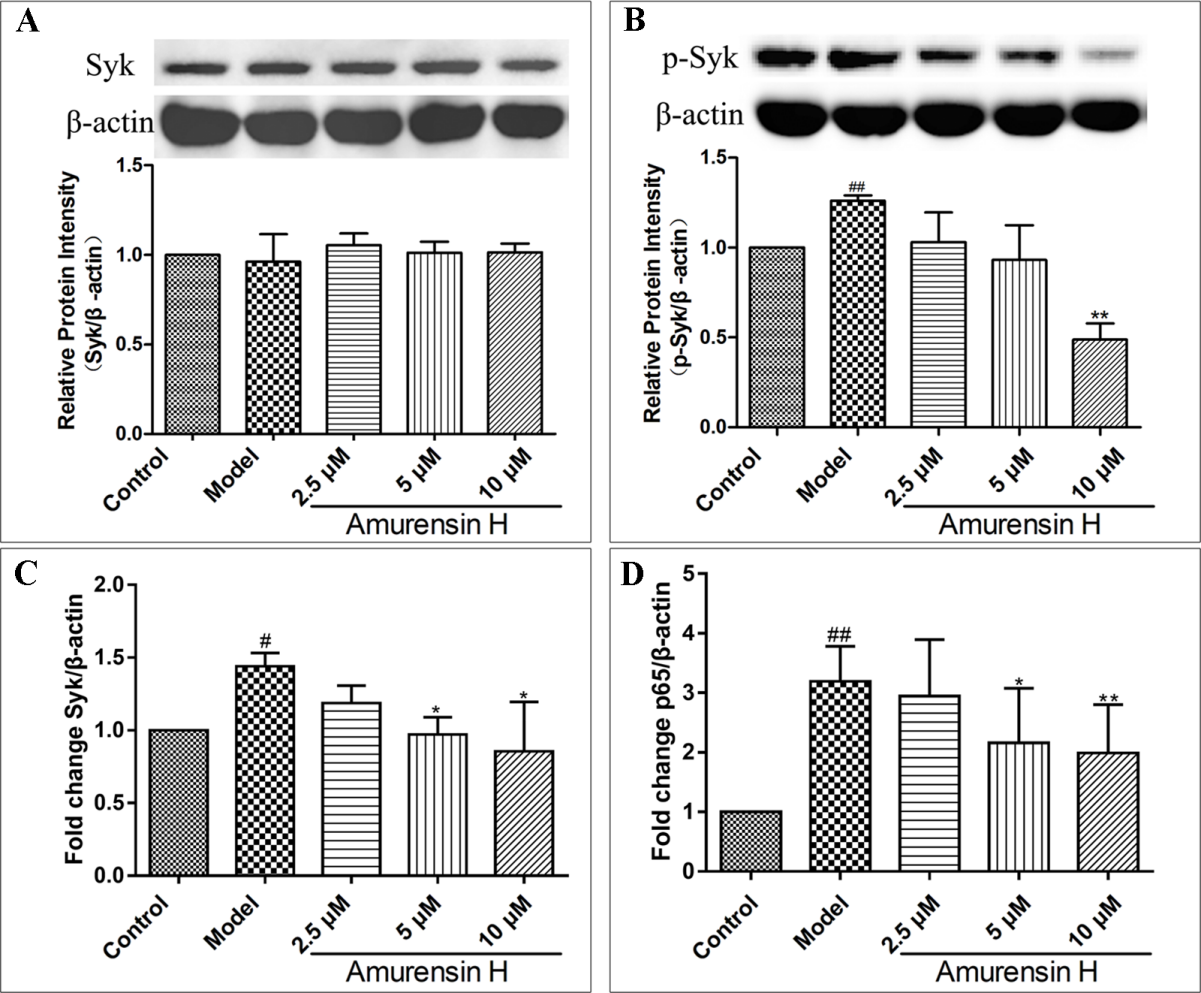
Expression of Syk and p65 in LPS-stimulated THP-1–derived macrophages *in vitro* treated with vehicle, LPS (1 µg/ml), amurensin H (2.5, 5, 10 µM). Effects of amurensin H on Syk protein expression **(A)** and p-Syk (Y323) protein expression **(B)** in THP-1–derived macrophages were detected by Western blotting. Effects of amurensin H on Syk mRNA expression **(C)** and p65 mRNA expression **(D)** in THP-1–derived macrophages were detected by quantitative polymerase chain reaction. Data are shown as mean ± SD of three to four independent experiments. ^#^ *P* < 0.05 and ^##^ *P* < 0.01 compared with the Control group; *P < 0.05 and **P < 0.01 compared with the Model group.

Coinciding with the Syk protein expression *in vivo*, amurensin H treatment notably suppressed the phosphorylation of Syk (**Figure 7B**), while the expression of Syk showed no statistical significance (**Figure 7A**) (**Supplementary Figures S5**, **S6**). However, as shown in **Figure 7C**, amurensin H treatment significantly inhibited the expression of Syk mRNA. Collectively, the results suggest that amurensin H treatment exhibited its anti-inflammatory effect *in vitro* by inhibiting Syk mRNA transcription and protein phosphorylation, but not by influencing Syk protein expression, which might associate with the process of translation of Syk mRNA into protein. Additionally, amurensin H treatment significantly inhibited p65 mRNA expression (**Figure 7D**).

### Amurensin H–Attenuated LPS-Stimulated Inflammation *in Vitro* Is Associated With the Inhibition of the Syk/NF-**κ**B Pathway

**Figure 8:**
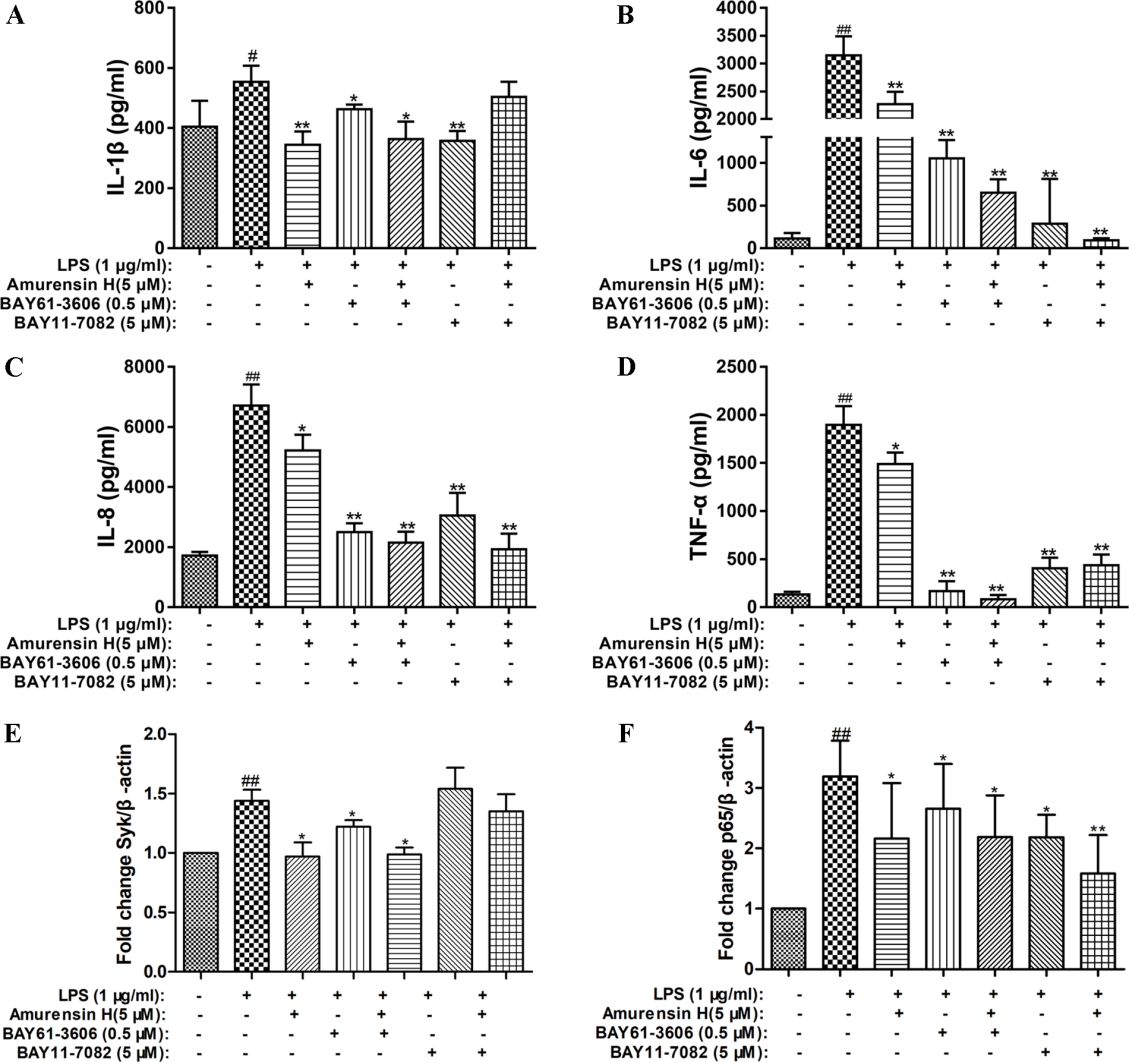
Verification that Syk and NF-κB were associated with amurensin H’s anti-inflammation activity in THP-1–derived macrophages. LPS-stimulated THP-1–derived macrophages were treated with vehicle, LPS (1 µg/ml), amurensin H (5 µM), BAY61-3606 (an inhibitor of Syk, 0.5 µM), and BAY11-7082 (an inhibitor of NF-κB, 5 µM). Levels of IL-1β **(A)**, IL-6 **(B)**, IL-8 **(C)**, and TNF-*α* **(D)** were detected by ELISA. The Syk mRNA expression **(E)** and p65 mRNA expression **(F)** were detected by quantitative polymerase chain reaction. Data are shown as mean ± SD of three to four independent experiments. ^#^ *P* < 0.05 and ^##^ *P* < 0.01 compared with the Control group; *P < 0.05 and **P < 0.01 compared with the Model group.

An inhibitor of Syk (BAY61-3606) and an inhibitor of its downstream NF-κB (BAY11-7082) were employed to verify the role of the Syk/NF-κB pathway in the protective process of amurensin H on airway inflammation. Treatment with amurensin H, BAY61-3606, and BAY11-7082 significantly reduced the production of IL-1β (**Figure 8A**), IL-6 (**Figure 8B**), IL-8 (**Figure 8C**), and TNF-α (**Figure 8D**) in LPS-stimulated THP-1–derived macrophages. The production of these cytokines was almost collaboratively inhibited by cotreating with amurensin H and BAY61-3606, while a substantial inhibition of IL-6 and IL-8 was achieved by cotreating with amurensin H and BAY11-7082. Interestingly, the Syk inhibitor (BAY61-3606) lessened the expression of Syk mRNA, which was made more effective by cotreating with amurensin H, but the NF-κB inhibitor did not affect Syk mRNA expression (**Figure 8E**). Furthermore, the Syk and NF-κB inhibitors both inhibited the expression of NF-κB mRNA, and both inhibition processes were enhanced by cotreatment with amurensin H (**Figure 8F**). Collectively, these data suppose that Syk/NF-κB may be a potentially important target through which amurensin H mediates COPD inflammatory response.

## Discussion

Cigarette smoke is the most important etiologic factor for COPD, and bacterial infections in the lungs are known to result in COPD exacerbations. Correspondingly, to assess the effect of amurensin H against COPD inflammation *in vivo* and *in vitro*, we herein used mice coexposed to CS and LPS to mimic airway inflammation of COPD exacerbations *in vivo* and used LPS stimulated THP-1–derived macrophages to mimic the acute inflammatory responses involved in COPD *in vitro*. The results in this study showed that amurensin H could notably alleviate the inflammatory processes and reduce inflammatory responses *in vivo* and *in vitro*. Amurensin H significantly reduced inflammatory cell infiltration in H&E-stained lung sections, as well as the number of alveolar macrophages and neutrophils in BALF of LPS/CS-induced mice. Furthermore, amurensin H–treated mice considerably exhibited lower levels of IL-6, IL-17A, TNF-α, and IFN-γ in BALF. Th1 cells produce IFN-γ, while IL-4 is produced by Th2 cells, a decline of IFN-γ/IL-4 could also be observed in different-dosage–treated LPS/CS-induced mice. In addition, amurensin H not only reduced the levels of inflammatory cytokines *in vivo*, but also decreased the production of IL-1β, IL-6, IL-8, and TNF-α in LPS-stimulated THP-1–derived macrophages *in vitro*.

To further extend these findings, we also studied potential mechanisms of amurensin H here. According to our previous study, amurensin H is an ATP-competitive inhibitor of Syk *in vitro* (Jiang et al., 2014). Activated Syk plays a critical role in macrophages, neutrophils, and airway epithelium inflammation; activating multiple inflammatory downstream signaling, including NF-κB pathway; and then regulating proinflammatory cytokine gene expression, such as TNF and IL-1β (Mocsai et al., 2002; Ulanova et al., 2007; Hilgendorf et al., 2011; Lu et al., 2014). Furthermore, it reported that Syk inhibitors could effectively alleviate airway inflammation of COPD patients during acute exacerbation (Angata et al., 2013). Therefore, Syk could be an attractive target for COPD airway inflammation because of its profound effects on allergic, inflammatory, and autoimmune diseases. Cigarette smoke–induced oxidative stress activates the transcription of activation of NF-κB, a downstream of Syk (Rajendrasozhan et al., 2008). Once activated, NF-κB complex enters the nucleus, thus regulating immune response and inflammation and regulating the expression of inflammatory mediators, including cytokines, chemokines, and cell adhesion molecules (**Supplementary Figure S7**) (Schuliga, 2015). The results of this study showed that the expression of p-Syk was significantly suppressed *in vivo* with amurensin H treatment, while the expression of Syk was not influenced. But, Syk mRNA levels were significantly decreased in amurensin H–treated THP-1–derived macrophage inflammation *in vitro*. This indicated that amurensin H exerted its anti-COPD airway inflammatory effect, possibly by inhibiting the phosphorylation and activation of Syk. Amurensin H inhibited the transcription of Syk gene but had no inhibition on protein expression of Syk, which may be associated with the regulation of Syk protein translation. Amurensin H may suppress the phosphorylation of tyrosine residues, which arouse kinase activation. This suppression probably also hinders transcription of Syk mRNA and influences arrangements of Syk protein in some way. We speculate that downregulation of Syk mRNA also is likely to associate with function of regulatory proteins, which regulate translation and rearrangement of Syk protein. However, the specific mechanisms of amurensin H on Syk are still pending and need to be studied further in future studies. We speculated that amurensin H may affect the transcription of Syk gene in two ways. First, amurensin H may impede the binding of transcriptional proteins to Syk gene; second, and likely more important, Syk gene may be bound by phosphorylated Syk, which is activated by amurensin H. Furthermore, amurensin H treatment significantly inhibited the expression of NF-κB p65 and p-NF-κB p65 in LPS/CS-induced mice, same as the expression of p65 mRNA *in vitro*, whereas expression of p65 and p-p65 protein with DEX, roflumilast, or resveratrol treatment showed no significant changes *in vivo*. As such, these results indicate that amurensin H might affect the Syk/NF-κB pathway to attenuate COPD airway inflammation.

In order to demonstrate the possibility that amurensin H exerted its anti-COPD inflammation function might be due to the inhibition of Syk/NF-κB pathway, Syk inhibitor (BAY61-3606) and NF-κB inhibitor (BAY11-7082) were chosen. The results showed that by cotreating with BAY61-3606 in LPS-stimulated THP-1–derived macrophages, amurensin H treatment superimposed the inhibition of IL-1β, IL-6, IL-8, and TNF-α production, whereas by cotreating with BAY11-7082, amurensin H treatment superimposed the inhibition of IL-6 and IL-8 production. Furthermore, by cotreating with BAY61-3606, amurensin H treatment superimposed the expression of Syk and NF-κB mRNA, while by cotreating with BAY11-7082, amurensin H treatment superimposed only the expression of NF-κB mRNA, but did not affect the expression of Syk mRNA. Syk is an essential enzyme in the immune system and is partly regulated through autophosphorylation, participating in Syk-mediated signal transduction (Mócsai et al., 2010). The results of this study also suggest NF-κB is a downstream molecule of Syk. Therefore, we speculate that amurensin H affects the activity of Syk through phosphorylation and then affects the activity of downstream NF-κB. However, mechanisms of amurensin H on Syk transcription have not been fully elucidated, the interaction of amurensin H and Syk transcription factor has not been studied in-depth in this study. Taken together, these results suggest that amurensin H exerted its anti-COPD inflammation effect partly through inhibition of expression of p-Syk and activation of NF-κB to modulate Syk/NF-κB pathway.

In conclusion, the present study revealed that amurensin H could ameliorate LPS/CS-induced airway inflammation *in vivo* and *in vitro*, suggesting that amurensin H could potentially be useful in COPD airway inflammation treatment, and inhibiting the Syk/NF-κB pathway might be part of its mechanism of action. Although further studies are warranted to evaluate the mechanisms by which amurensin H mediates Syk/NF-κB in COPD disease, some of the limitations of this study are addressed in that i) LPS/CS-induced mice only have relevance to inflammation found in COPD; long-term COPD models are needed to test the anti-inflammatory effect of amurensin H and potential mechanisms; ii) the effect of amurensin H on Syk transcription *in vitro* was not determined. To explore its specific effect, we will use Syk shRNA in future studies to inhibit the expression of Syk mRNA as the reference group, explore amurensin H’s effect on Syk mRNA and Syk protein in combination with Syk shRNA, and test how long amurensin H took to deplete Syk protein in cell lines. In further studies, genomics analysis (such as RNA-seq) also will be used. iii) Our findings have clearly demonstrated that the effect of amurensin H may represent a novel approach to treating inflammation; combined use of amurensin H and dexamethasone may be a method to be examined by which to ameliorate inflammation in COPD disease.

## Data Availability Statement

The datasets generated for this study are available on request to the corresponding author.

## Ethics Statement

The animal study was reviewed and approved by the institutional guidelines at the Experimental Animal Center of the Institute of Materia Medica, Chinese Academy of Medical Sciences & Peking Union Medical College.

## Author Contributions

ML and QH conceived and planned the experiments. YaF, JB, HY, SL, and JY performed animal experiments. YaF and ZZ performed cellular experiments. CY provided amurensin H and instructed YiF to analyze its purity. YaF processed the experimental data, performed the analysis, drafted the manuscript, and designed the figures with support from ML and QH. PM, ZZ, and ML contributed to the final version of the manuscript. All authors discussed the results and commented on the manuscript.

## Funding

This work was financially supported by the Beijing Natural Science Foundation Program (grant no. 7182116), the National Natural Science Foundation of China (grant no. 81973539, 81473398, and 81603359), the CAMS Initiative for Innovative Medicine (grant no. 2016-I2M-2-006), and the Drug Innovation Major Project of China (grant no. 2018ZX09711001-003-001). The funders had no role in study design, data collection and analysis, decision to publish, or preparation of the manuscript.

## Conflict of Interest

The authors declare that the research was conducted in the absence of any commercial or financial relationships that could be construed as a potential conflict of interest.

## Abbreviations

COPD, chronic obstructive pulmonary disease; CS, cigarette smoke; LPS, lipopolysaccharide; IL, interleukin; BALF, bronchoalveolar lavage fluid; NF-κB, nuclear factor κB; SH2, Src homology 2; ITAM, immunoreceptor tyrosine-based activation motif; ELISA, enzyme-linked immunosorbent assay.
