# Supplementary material for: Amurensin H, a Derivative From Resveratrol, Ameliorates Lipopolysaccharide/Cigarette Smoke–Induced Airway Inflammation by Blocking the Syk/NF-κB Pathway
Source: Front Pharmacol. 2019 Oct 4;10:1157. doi: 10.3389/fphar.2019.01157 (PMC6787933; doi:10.3389/fphar.2019.01157)

**Supplementary figures**


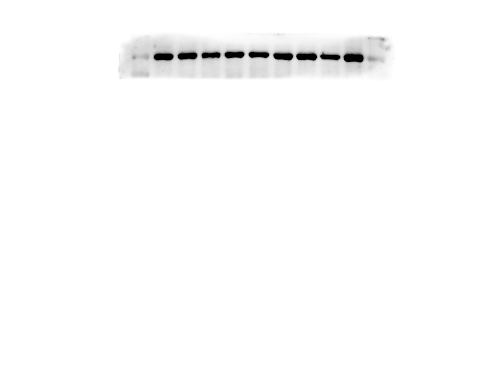


S1-Syk


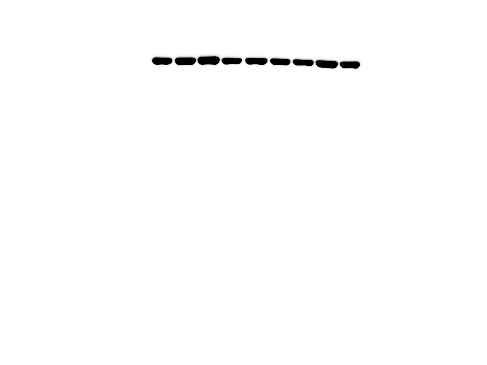


S1 β-actin

Figure S1. Full, untruncated images of Figure 5A (Control-Model- Amurensin H 5 mg/kg - Amurensin H 10 mg/kg-Amurensin H 20 mg/kg-DEX-RFST-RES-useless control).


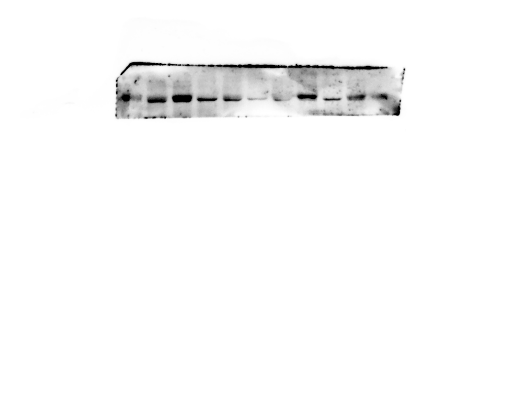


S2 p-Syk


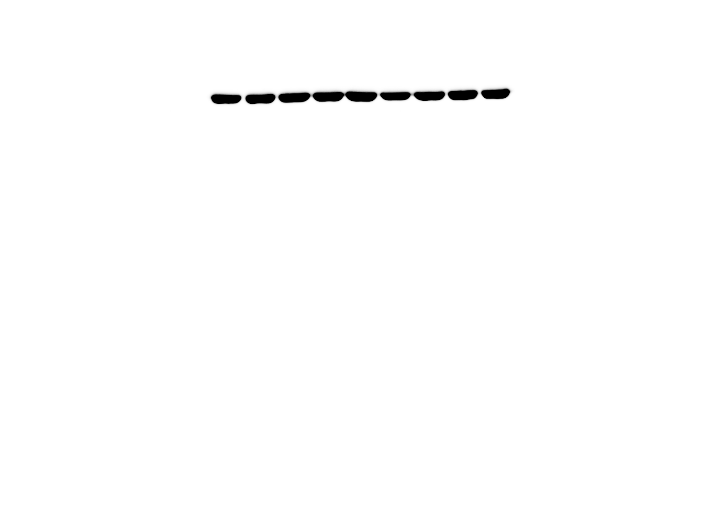


S2 β-actin

Figure S2. Full, untruncated images of Figure 5B (Control-Model- Amurensin H 5 mg/kg - Amurensin H 10 mg/kg-Amurensin H 20 mg/kg-DEX-RFST-RES-useless control)


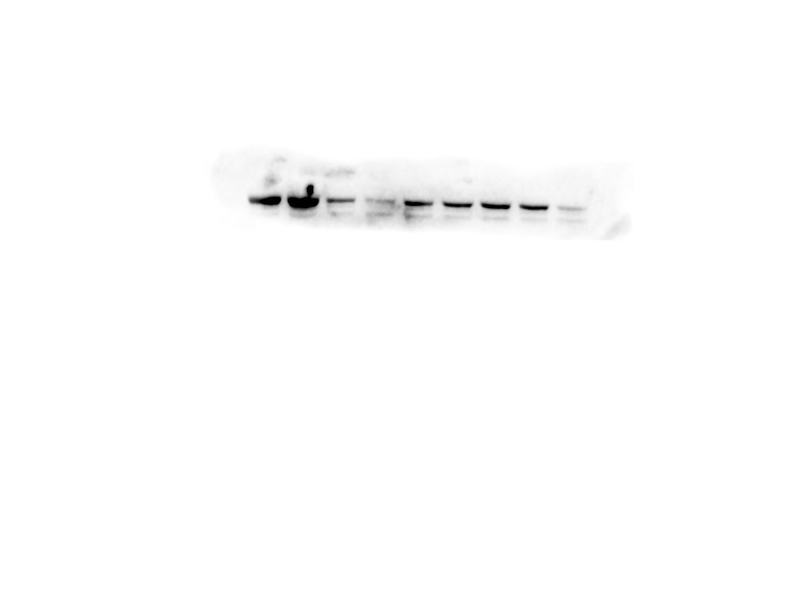


S3 p65


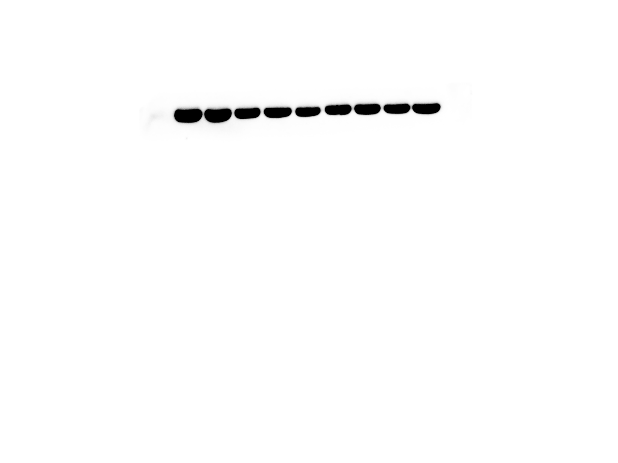


S3 β-actin

Figure S3. Full, untruncated images of Figure 5C (Control-Model- Amurensin H 5 mg/kg - Amurensin H 10 mg/kg-Amurensin H 20 mg/kg-DEX-RFST-RES-useless control).


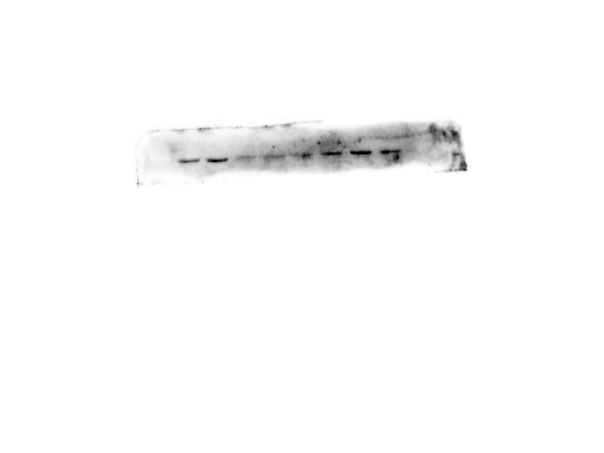


S4 p-p65


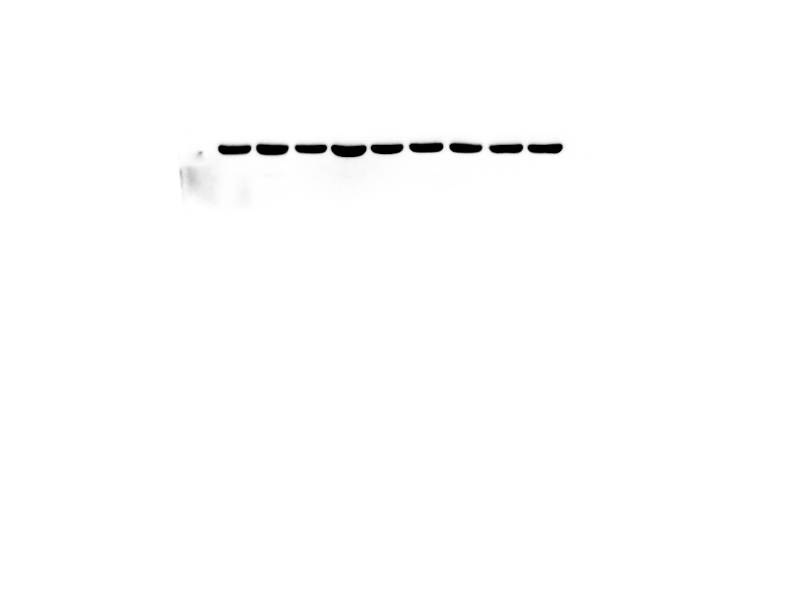


S4 β-actin

Figure S4. Full, untruncated images of Figure 5D (Control-Model- Amurensin H 5 mg/kg - Amurensin H 10 mg/kg-Amurensin H 20 mg/kg-DEX-RFST-RES-useless control).


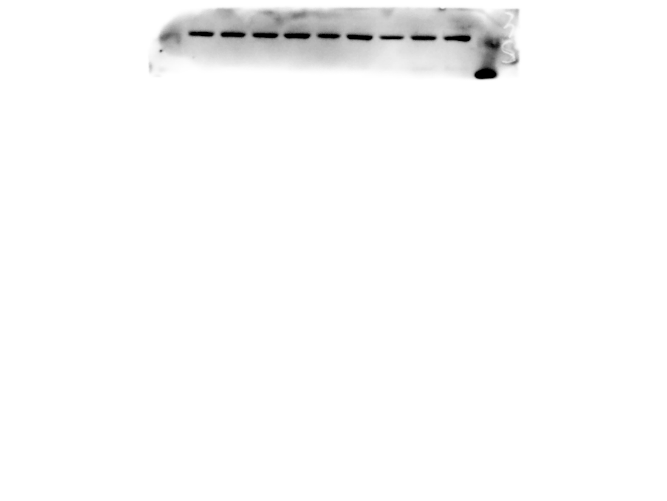


S5 Syk


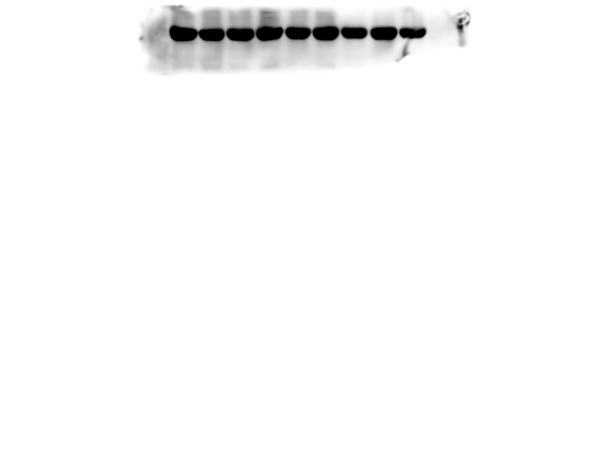


S5 β-actin

Figure S5. Full, untruncated images of Figure 7A (Control-Model- Amurensin H 2.5mM- Amurensin H 5mM -Amurensin H 10mM- useless control - useless control - useless control -useless control)


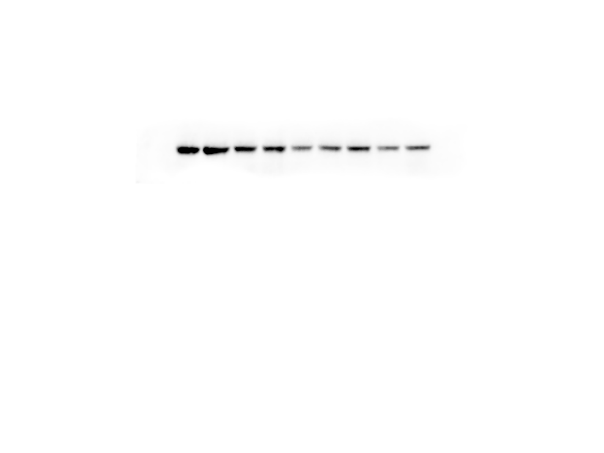


S6 p-Syk


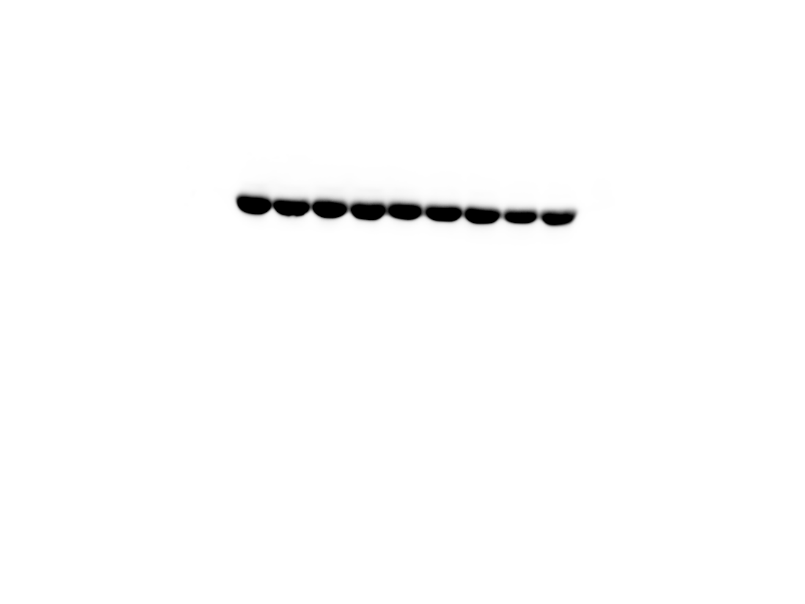


S6 β-actin

Figure S6. Full, untruncated images of Figure 7B (Control-Model- Amurensin H 2.5mM- Amurensin H 5mM -Amurensin H 10mM- useless control - useless control - useless control -useless control).


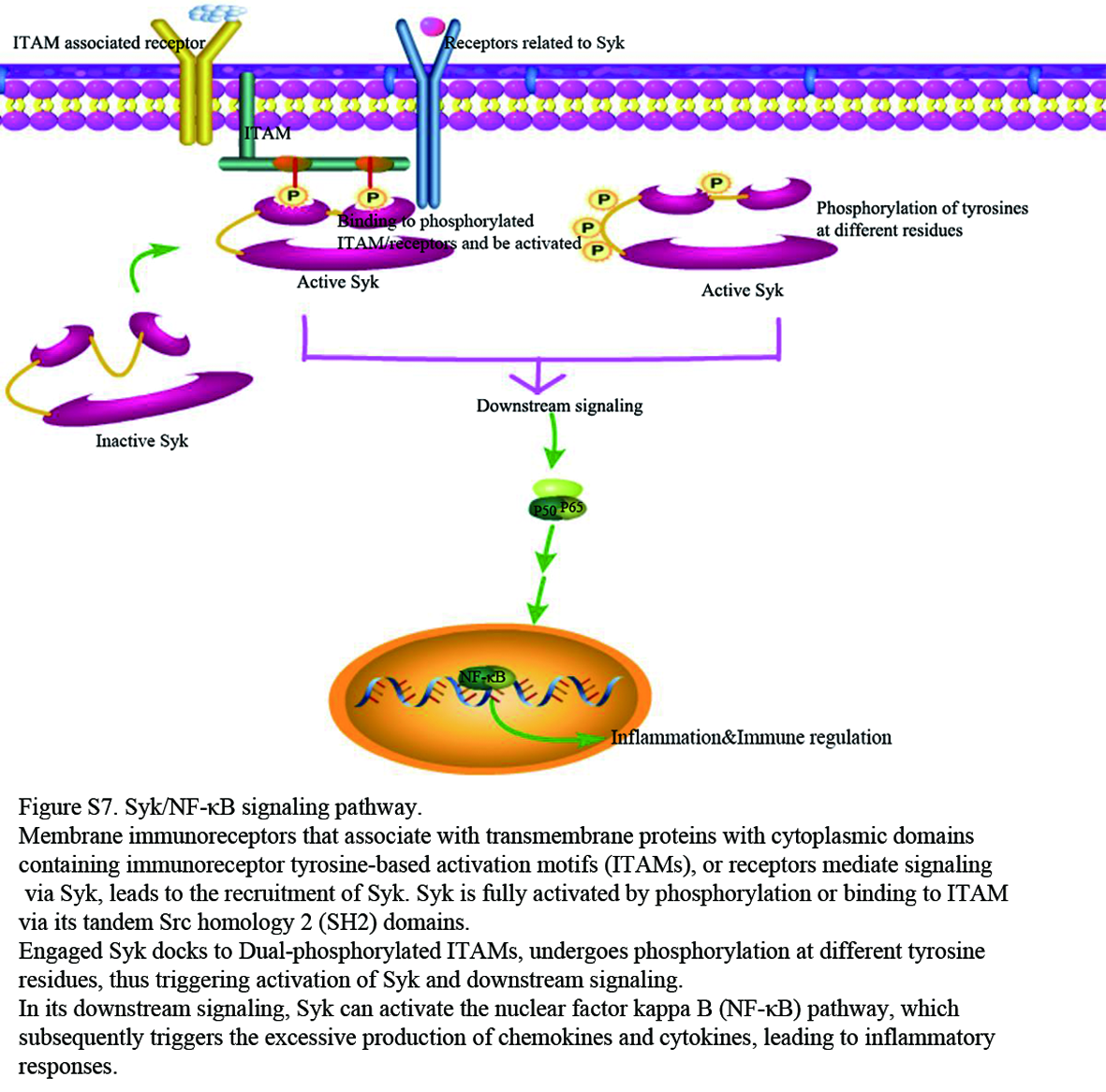

Supplement: Supplementary file 1 [file DataSheet_1.docx]
